# Performance of Mattis dementia rating scale-Chinese version in patients with mild cognitive impairment and Alzheimer’s disease

**DOI:** 10.1186/s12883-021-02173-0

**Published:** 2021-04-21

**Authors:** Shuxia Qian, Keliang Chen, Qiaobing Guan, Qihao Guo

**Affiliations:** 1 Department of Neurology, The Second Affiliated Hospital of Jiaxing University, Jiaxing, China; 2 Department of Neurology, Huashan Hospital, Fudan University, Shanghai, China; 3 Department of Gerontology, Shanghai Jiao Tong University Affiliated Six People’s Hospital, Shanghai, China

**Keywords:** Alzheimer’s disease, Mild cognitive impairment, Mattis dementia rating scale

## Abstract

**Background:**

To identify the applicability of the Chinese Version of Mattis Dementia Rating Scale (DRS-CV).

**Methods:**

The DRS-CV was administered to 483 participants, including 136 normal controls, 167 patients with mild cognition impairment (MCI), and 180 patients with Alzheimer’s disease (AD). Receiver Operating Characteristic (ROC) curve was used to evaluate the sensitivity and specificity of the scale.

**Results:**

The scores of DRS-CV were ranked in the order of NC > MCI > mild AD > moderate AD group. Memory was the sensitive function affected at a relatively earlier stage of AD. ROC curve analysis indicated the DRS-CV total score and memory subscale showed excellent sensitivity and specificity in the discrimination between MCI from mild AD and mild AD from moderate AD, but poor sensitivity and specificity in the discrimination between MCI and NC.

**Conclusion:**

The DRS-CV is useful to the early diagnosis and severity of AD, not to the early identification of MCI.

**Supplementary Information:**

The online version contains supplementary material available at 10.1186/s12883-021-02173-0.

## Background

The Mattis Dementia Rating Scale (DRS) was used as a screening tool to assess the cognitive function for patients with dementia. It consists of 37 tasks divided into five subscales of attention, initiation/perseveration, construction, conceptualization, and memory, with subscale scores of 37, 37, 6, 39, 25, respectively and a maximum total score of 144.

The DRS provides more detailed information about a patient’s impaired and intact cognitive function than other widely used mental state examination tools, such as the Mini-mental state examination (MMSE). The pattern of DRS scores obtained on the five domains can show qualitative differences in the cognitive profiles of different types of dementia, including Parkinson’s disease (PD) [1], Huntington’s disease [2], Lewy Body dementia [3] and Progressive supranuclear palsy [4]. Recently, the scale has been appled to a wider range disorders, such as mild cognitive impairment (MCI) due to PD, and schizophrenia [5].

Some sociodemographic factors, such as age and education, have been found to have a significant impact on the performance of DRS [6], and cross-cultural comparisons of the performance of instruments are often needed before generalisations can be made to apply them to people from different cultures. The scale was translated into Chinese, and the test-retest and inter-rater reliability of Chinese Version of the Dementia Rating Scale (DRS-CV) was excellent [7]. Since the DRS-CV has not yet been adoped nationwide, relavant studies have been conducted only in Hong Kong [8, 9]. The study sample was relatively small and the identification of cognitive impairment was not detailed. Our study, for the first time, validates Mandarin Chinese version of DRS and further assesses the applicability of DRS. 

## Methods

### Participants

One hundred and thirty-six normal controls (NC) were recruited from the urban centers of Jingansi district of Shanghai. Controls had no known neurological or psychiatric diseases and were designated to be normal on the Clinical Dementia Rating Scale (CDR = 0) [10] and Mini-mental state examination-Chinese version (CMMSE score>cutoff) [11].

One hundred and sixty-seven patients with MCI and one hundred and eighty patients with AD were recruited from Memory Clinic of Huashan Hospital.

The inclusion criteria for MCI [12] included the following: (1) cognitive impairment verified by an agent or caregiver; (2) symptoms last for more than 3 months; (3) between the ages of 50 and 90; (4) the lenth of schooling from 2 to 18 years; (5) the total score of CMMSE>cutoff [11], abnormal objective cognitive impairment documented by scoring below the age and education adjusted cutoff on the Neuropsychological assessments (Auditory verbal learning test, et al) [13], preserved basic activities of daily living/minimal impairment in complex instrumental functions; (6) unknown etiology, (7) normal sense of hearing and sight; (8) dementia diagnostic criteria that do not meet the diagnostic criteria of the national institute on aging (NIA) and the alzheimer’s association (AA) [14]. 152 patients amnestic-MCI and 15 patients with nonamnestic MCI were recruited.

The diagnosis of AD is based on the NIA-AA standard diagnostic guidelines [14]. All patients were screened in detail including the history of brain disease, physical illness, and mental states such as anxiety and depression. In addition, all patients underwent neurological examinations, thyroid function tests (FT_3_, FT_4,_ and TSH), folic acid, and vitamin B12 measurements. CT or MRI scan of brain were conducted for all patients to exclude vascular factors (including lacunar infarctions or diffuse white matter ischemic changes). According to CDR, there were 116 cases of mild dementia (CDR = 1) and 64 cases of moderate dementia (CDR = 2) [10].

This study was approved by the ethics committee of Huashan Hospital, and written consent was obtained from subjects or their legally authorized caregivers.

### Neuropsychological assessment

The DRS-CV and CMMSE were used. The CDR was supported by participant and caregiver interview. Meanwhile, participants from Memory Clinic were also administered a battery of neuropsychological tests, including Auditory verbal learning test (AVLT),^14^ Logical memory test (LMT),^15^ Rey-Osterrieth Complex Figure Test (CFT),^16^ Clock-drawing test (CDT),^17^ Verbal fluency test (VFT),^18^ Stroop color-word test (SCWT),^19^ Trail making test (TMT),^20^ and Center for Epidemiology Scale-Depression (CES-D).^21^ All neuropsychological battery was administered by clinical psychologists or specially trained doctoral level students who were blinded to diagnosis.

### Statistical analysis

The χ2 test or one-way analysis of variance was used to test for the differences among the four groups (NC, MCI, mild AD and moderate AD groups) in the distribution of gender, age, education level and performance on neuropsychological tests. Bivariate correlation were applied to analyze the relation between two variables. Receiver Operating Characteristic (ROC) curve was used to evaluate the sensitivity and specificity of the scale. The level of significance was set at α = 0.05. All analyses were conducted using SPSS version 19.0.

## Results

### Demographic characteristics and performance of CMMSE and DRS-CV

Demographic information and the scores of CMMSE and DRS-CV among four groups including NC, MCI, mild AD and moderate AD were presented in Table 1. No difference was found in age (F = 2.428, *p* = 0.065), sex distribution (χ2 = 0.647, *p* = 0.886) and education (F = 1.500, *p* = 0.214) among the four groups.

**Table 1:** Demographic information and performance of CMMSE and DRS among four groups

	NC (*n* = 136)	MCI (*n* = 167)	Mild AD (*n* = 116)	Moderate AD (*n* = 64)	P
Age (year)	68.74 ± 8.93	68.81 ± 8.30	71.29 ± 9.14	70.27 ± 9.24	0.065
Sex (Male/Female)	65/71	86/81	59/57	34/30	0.886
Education (year)	10.73 ± 3.89	11.21 ± 3.39	10.53 ± 4.03	10.16 ± 3.76	0.214
CMMSE total score (max = 30)	28.20 ± 2.10^*†‡^	26.87 ± 1.93^§¶^	21.66 ± 2.68^#^	13.37 ± 2.25	< 0.001
DRS total score (max = 144)	133.71 ± 6.64^*†‡^	128.59 ± 7.03^§¶^	110.43 ± 10.71^#^	91.20 ± 17.42	< 0.001
Attention (max = 37)	36.51 ± 0.76^†‡^	36.14 ± 1.26^§¶^	35.14 ± 2.00^#^	32.66 ± 3.85	< 0.001
Initiation/Perseveration (max = 37)	32.50 ± 4.12^†‡^	31.54 ± 5.04^§¶^	23.40 ± 6.03^#^	17.05 ± 6.77	< 0.001
Construction (max = 6)	5.86 ± 0.65^†‡^	5.68 ± 0.74^§¶^	5.16 ± 1.35^#^	3.86 ± 1.79	< 0.001
Conceptualization (max = 39)	35.68 ± 2.82^*†‡^	34.26 ± 2.99^§¶^	31.53 ± 4.34^#^	27.25 ± 7.66	< 0.001
Memory (max = 25)	23.16 ± 2.03^*†‡^	20.97 ± 2.89^§¶^	15.21 ± 3.87^#^	10.39 ± 3.93	< 0.001

Notes: *AD* Alzheimer’s disease, *MCI* mild cognitive impairment, *CMMSE* mini-mental state examination-Chinese version, *DRS* Mattis Dementia Rating Scale, *NC* normal controls

^*^ *p* < 0.05, NC vs. MCI; ^†^ *p* < 0.05, NC vs. Mild AD; ^‡^ *p* < 0.05, NC vs. Moderate AD; ^§^*p* < 0.05, MCI vs. Mild AD; ^¶^ *p* < 0.05, MCI vs. Moderate AD; ^#^ *p* < 0.05, Mild AD vs. Moderate AD

The total scores of CMMSE and DRS-CV were significantly lower in patient groups. The scores of different cognitive domains were ranked in the order of NC > MCI > mild AD > moderate AD group. Subscales of conceptualization and memory was affected at a relatively earlier stage.

### Correlations between demographic variables and DRS total and subscale scores

We analyzed the effects of age, sex and education on DRS-CV total and subscale scores for NC. There was no difference on DRS-CV total and subscale scores between male and female (all *p* > 0.05). Correlation analysis revealed that age was correlated to DRS-CV total score (r = − 0.264, *p* = 0.002) and initiation/perseveration score (r = − 0.223, *p* = 0.009), and education was correlated to DRS-CV total score (r = 0.254, *p* = 0.003), attention score (r = 0.363, *p* < 0.001) and initiation/perseveration score (r = 0.271, *p* = 0.001) (Table 1).

### ROC analysis of DRS-CV total and subscale scores for discriminating MCI from NC, MCI from mild AD and mild AD from moderate AD (Supplementary Figure 1)

The most appropriate cutoffs of DRS-CV were 131 in the discrimination between MCI and NC (sensitivity 65.3%, specificity 67.6%, AUC 0.708), 120 in the discrimination between MCI and mild AD (sensitivity 84.5%, specificity 85.0%, AUC 0.924), 103 in the discrimination between mild and moderate AD (sensitivity 79.7%, specificity 78.4%, AUC 0.846) (Table 2). Using these cutoff scores, the DRS-CV showed excellent sensitivity and specificity in the discrimination between MCI from mild AD and mild AD from moderate AD, but poor sensitivity and specificity in the discrimination between MCI and NC, suggesting that the predictive information captured by the DRS total score was only reasonably good to detect AD but not MCI.

**Table 2:** ROC analyses of DRS total and subscale scores for discriminate MCI from NC, MCI from mild AD and mild AD from moderate AD

	NC vs. MCI					MCI vs. Mild AD				Mild AD vs. Moderate AD			
	AUC	cut-off score	Sensitivity (%)	Specificity (%)	AUC	cut-off score	Sensitivity (%)	Specificity (%)	AUC	cut-off score	Sensitivity (%)	Specificity (%)	
DRS total score	0.708	131	65.3	67.6	0.924	120	84.5	85.0	0.846	103	79.7	78.4	
Attention	0.581	36	48.5	65.4	0.681	36	75.9	51.5	0.740	34	59.4	76.7	
Initiation/perseveration	0.545	30	38.9	72.1	0.845	29	87.1	67.7	0.756	16	50.0	87.9	
Construction	0.565	5	20.4	92.6	0.605	5	39.7	79.6	0.720	4	56.2	79.3	
Conceptualization	0.639	36	80.8	44.1	0.696	32	53.4	83.8	0.670	32	78.1	46.6	
Memory	0.7434	22	68.3	72.8	0.885	19	87.1	77.2	0.823	13	84.4	72.4	

*Notes*: *AD* Alzheimer’s disease, *DRS* Mattis Dementia Rating Scale, *MCI* mild cognitive impairment, *NC* normal controls

The memory subscale showed good sensitivity and specificity in the discrimination between MCI from mild AD and mild AD from moderate AD, similar to the DRS total score. In comparison, the AUC of the memory subscale for discriminating MCI from mild AD was similar to the initiation/perseveration subscale (0.885 versus 0.845, *p* = 0.201) and larger than other three subscales (attention: 0.885 versus 0.681, *p* < 0.001; construction: 0.885 versus 0.605, p < 0.001; conceptualization: 0.885 versus 0.695, p < 0.001). For discriminating mild AD from moderate AD, the AUC of the memory subscale was lager AUC than other four subscales, but not significant (attention: 0.823 versus 0.740, *p* = 0.100; initiation/perseveration: 0.823 versus 0.755, *p* = 0.179; construction: 0.823 versus 0.720, *p* = 0.051; conceptualization: 0.823 versus 0.670, *p* = 0.005). All DRS-CV subscale scores had poor sensitivity and specificity for discriminating MCI from mild AD.

## Discussion

Screening for AD is an important clinical necessity for early diagnosis and initiating proper treatment. The DRS is a psychometric instrument designed to assess the nature and severity of dementia. Five subscales are included to evaluate different domains of cognitive function.

In our study, we evaluated the performance of DRS-CV in different stages of AD, including very early stage (MCI). At the early stage of dementia, memory and conceptualization were first impaired. With progression of the disease, other functions such as initiation/perseveration and attention may be affected. ROC curve analysis indicated DRS-CV could be administered to differentiate AD from MCI, but was not effective for detecting MCI, which was inconsistent with previous research. Matteau et al. reported that the DRS was useful to detect and differentiate between patients with amnestic MCI. The reasons may be the instability of the DRS to identify cognition impairment and the differences in lifestyle and culture. Meanwhile, it is important to establish whether each of the five DRS subscales contributes equally to the diagnostic power. The result of ROC analysis showed the memory subscale might be better than other four subscales.

To our knowledge, several studies explored the effect of age and education on the DRS-CV score. Age and education conversion scores were obtained from researchers associated with the Mayo Older American Normative Studies (MOANS) [15]. Five equations to adjust for the age and educational level of the scores were provided [9]. In our study, age and educational level were related to the performance of DRS, consistent with previous studies.

We found the initiation/perseveration score of NC sample in our study was inferior to the studies in western countries and Hong Kong. Possible differences in lifestyle and culture might account for those differences. Initiation/perseveration subscale of DRS-CV included five tests: Supermarket Fluency, Clothing Fluency, Verbal Repetition, Double Alternating, and Graphomotor. For example, supermarkets were not ubiquitous in most cities of China until recent years, which may be a reason for low scores of that test in the Chinese elderly population.

Overall, our study verified the applicability of DRS-CV to detect AD but not MCI. Our study was limited by several factors. There were no subjects with severe dementia (CDR = 3). The percentage of different stages was not evenly distributed. Subjects with low level of education and uneducated were not recruited adequately. Further investigation is required to confirm these results and to determine whether the cutoff scores are suitable in patients with lower educational level.

## Conclusion

The DRS-CV is useful to the early diagnosis and severity of AD, not to the early identification of MCI.

## Supplementary Information

**Additional file 1: Supplementary Figure 1.**

**Additional file 2.**

## Data Availability

The datasets used or analysed during the current study are available from the corresponding author Qihao Guo on reasonable request.

## Abbreviations

DRS: Mattis Dementia Rating Scale
DRS-CV: Chinese Version of the Dementia Rating Scale
AD: Alzheimer’s disease
MCI: mild cognitive impairment
ROC: Receiver Operating Characteristic
MMSE: Mini-mental state examination
PD: Parkinson’s disease
NC: normal controls
CMMSE: Mini-mental state examination-Chinese version
AVLT: Auditory verbal learning test
LMT: Logical memory test
CFT: Rey-Osterrieth Complex Figure Test
CDT: Clock-drawing test
VFT: Verbal fluency test
SCWT: Stroop color-word test
TMT: Trail making test
MOANS: Mayo Older American Normative Studies
