# Supplementary figures and images for: Performance of Mattis dementia rating scale-Chinese version in patients with mild cognitive impairment and Alzheimer’s disease
Source: BMC Neurol. 2021 Apr 21;21:172. doi: 10.1186/s12883-021-02173-0 (PMC8059185; doi:10.1186/s12883-021-02173-0)

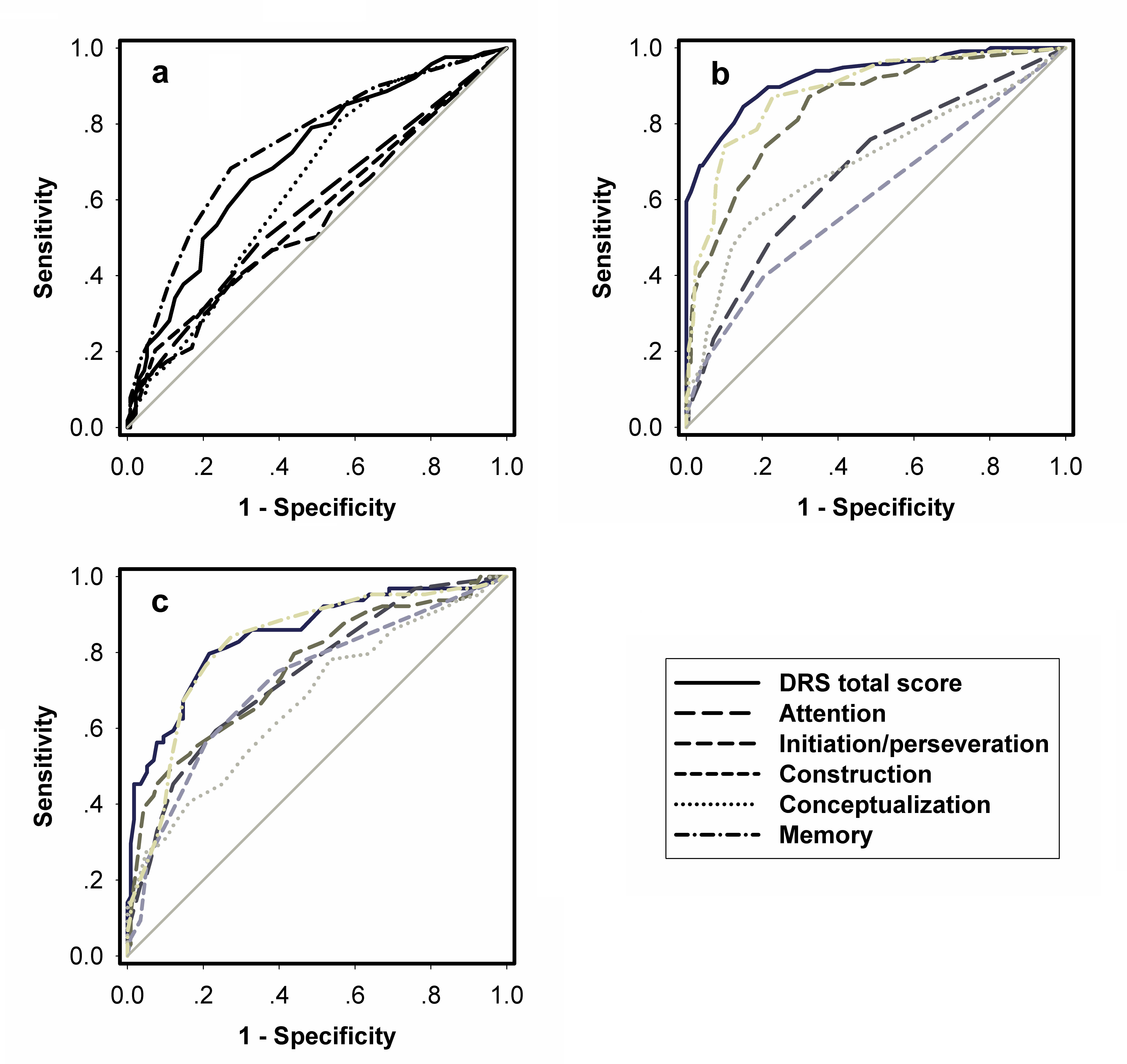

Supplement: Supplementary file 1 — Additional file 1: Supplementary Figure 1. [file 12883_2021_2173_MOESM1_ESM.tif]
