# Supplementary material for: Performance of Mattis dementia rating scale-Chinese version in patients with mild cognitive impairment and Alzheimer’s disease
Source: BMC Neurol. 2021 Apr 21;21:172. doi: 10.1186/s12883-021-02173-0 (PMC8059185; doi:10.1186/s12883-021-02173-0)
Supplement: Supplementary file 2 — Additional file 2. [file 12883_2021_2173_MOESM2_ESM.doc]

Cognitive Examination Record Form

[S](../../../../E:/Program%20Files%20(x86)/Youdao/Dict/8.9.6.0/resultui/html/index.html#/javascript:;)erial [number](../../../../E:/Program%20Files%20(x86)/Youdao/Dict/8.9.6.0/resultui/html/index.html#/javascript:;):

[N](../../../../E:/Program%20Files%20(x86)/Youdao/Dict/8.9.6.0/resultui/html/index.html#/javascript:;)ame [G](../../../../E:/Program%20Files%20(x86)/Youdao/Dict/8.9.6.0/resultui/html/index.html#/javascript:;)ender  Age （ year month ） Marriage：

Education level of interviewee:Years of formal schooling_______year

| one.No primary education | two. One add A private school or tutor | three. One add Adult literacy classes or evening classes | four.Primary industry spreading |
| --- | --- | --- | --- |
| five.finish primary school | six.Junior high school drop-outs or graduation | seven. High school boss industry or graduation | eight. College degree or above |

Pre-retirement occupation： left-hander or right-hander?: Date of assessment： assessment officer ：

Phone:  location:

Family history of stroke: History of disturbance of consciousness hypertension history History of diabetes The history of drinking history of epilepsy History of cranial trauma Other important medical history Neurological signs

**History of present illness**

**CT or MRI**

**Clinical diagnosis**

| one.What's the year of this year? ＿ year two.What season is it? ＿ season  three.What month is this?＿month four.What's the date today?＿date  five.what day is it today? ＿ six.Which province and city are we in now?＿  seven.What district (county) do you live in? district (county)＿ eight.What street do you live on? Street (Township)＿  nine.What floor are we on now? Floor＿ ten.Where are we? Address (Name)＿＿ |
| --- |
| eleven.Now I'm going to say the names of three things, and when I'm done, repeat them, and remember them, because I'll ask you again later: "The ball, the flag, the tree."(Score for the first answer). The ball＿the flag＿the tree＿ |
| twelve.Now subtract seven from one hundred, then subtract seven from the number you get, and so on, giving me each answer until I say, "Stop."(If a mistake is made, but the next answer is correct, then only one mistake is recorded.) ninety-three＿eighty-six＿seventy-nine＿seventy-two＿sixty-five＿ |
| thirteen.Now tell me, what are the three things I just asked you to remember?  The ball＿the flag＿the tree＿ |
| fourteen.(The questioner takes out his watch) What is this, please?  watch＿(Take out the pencils)What is this? pencils＿ |
| fifteen.Now I will say one word, repeat it clearly, and it is: "Forty-four stone lions."(Say it once only. Only if it is correct and clearly spoken will score one point.) |
| sixteen.(The interviewer hands the interviewee a card with the words "Close your eyes" written on it.)Please follow the instructions on the card.(If he closes his eyes, score one.) |
| seventeen.(The interviewer says the following paragraph and gives him a blank sheet of paper. Do not repeat the statement or demonstrate.)  Take the paper in your right hand＿Then fold the paper in half with both hands＿＿Place the paper on your lap＿ |
| Eighteen.Please say a complete, meaningful sentence (sentence must have a subject, a verb)  Write down the sentence＿ |
| nineteen.Please draw the picture as it looks. (painted on the back) |

**Mattis Dementia Rating Scale**

（Note: The results of A and W can be obtained in the following individual tests）

*A.digit span ：Please read some numbers after me. For example, if I say six or seven, you will tell mesix or seven. Pay attention to what I'm saying, and you won't start talking until I'm done.A1. Memorize in order two，five ______three，one，six_______

four，seven，nine，two________

score（zero，two，three，four）_____▲

I'll tell you some more numbers, and when I'm done, you'll have to turn them upside down. For example, when I say one to two, you say two to one... Do you understand?... Ready to start... A2. Memorize in order one，four______five，three，nine______eight，five，nine，three

Score（zero，two，three，four）__________▲

B．Two instructions in a row (full score for B=r2, full score for E，C-D)

I'll ask you to do something. Please do as I say.

B one. "Please open your mouth and close your eyes."（one point）________

B two. "Please stick out your tongue, and then raise your hand."（one point）________

Score B（zero to two） ___________▲

1. Single instruction: I will ask you to do some actions, please do as I say. One point per sentence.

C one. Open your mouth C two. Stick out your tongue C three. Close your eyes, C four. Raise your right hand.

Score C（zero to four） ___________▲

1. Imitate and look at me... Please do as I do.

D one. Open your mouth D two. Shake your head D three. Close your eyes, D four. Hands up

Score D（zero to four） ___________▲

E。The initiative / persistence of complex spoken English (if the score is E > 13, you will get a full score when you go to Imam Flyh) Please tell me everything you can buy in the supermarket and try to speak as much as possible until I tell you to stop. Start... (with a time limit of sixty seconds, you will get one point for each correct answer.)

| **One to fifteen seconds** | **Sixteen to thirty seconds** |
| --- | --- |
| **thirty-one to forty-five seconds** | **forty-six to sixty seconds** |

Score E（zero to twenty）___________●

F．Initiative / persistence of simple spoken English

**Please tell me the names of all the clothes. (with a time limit of sixty seconds, you will get one point for each correct answer.)**

| **One to fifteen seconds** | **Sixteen to thirty seconds** |
| --- | --- |
| **thirty-one to forty-five seconds** | **forty-six to sixty seconds** |

**Score F（zero-eight）___________●**

G。Consonant persistence **Please tell me "ben", "ken" and "gen". Then please say "ben-ken-gen" four times.**“ben-ken-gen”…**four times**（one point）___________

score G（zero to one）___________●

1. Vowel persistence  **Please tell me "b ei", "ba" and "bo". Then please say "bei-ba-bo"** **four times.**“bei-ba-bo” …**four times**（one point）___________ score H（zero to one）___________●

I. Double alternating action (if the score is I=one, full score will be given if you go to L,J to K)

Now I want you to do something with your hands. Watch me do it, and then do what I do. The palm of one hand is up, the palm of the other is down, and then turn around. You keep doing it until I tell you to stop. The palm of one hand is up, the palm of the other is down.five times (one point)

Score I___●

J.Double alternating movements Now do this... Make a fist with one hand, let go of the finger with the other, and then turn around. You keep doing it until I tell you to stop.

One hand clenched fist, the other let go of the finger. 5 times (one point)

Score J（zero to one）___●

K.Alternate tapping action. Now do this. Knock on the left, then on the right, then on the left, and then on the right. You keep doing it until I tell you to stop. Knock on the left, then on the right. ten times (one point)

Score K______●

L. Drawing design one (if the score L = one, go to P, M-O, give full points)

Explain the card one in the test tool booklet and give the subject a white paper.**Imitate this pattern (pointing from left to right, pointing to "barriers" pattern), please draw it on white paper.**

Score L（zero to one）______●

1. Drawing design two Finger in card two。

**Imitate this pattern ("round"in the finger, please draw it on this (finger in blank paper).**

Score M（zero to one）____●

1. Drawing design three Finger in card three。**Imitate this pattern**（**finger in**“X”），**please draw it on this blank paper)** Score N（zero to one）_____●

O. Drawing design four Finger in card four。**Imitate this pattern**（Finger with your finger "alternating XOXO pattern"），**please draw it on this blank paper** Score O（zero to one）_____●

1. Picture design one Finger in card five。**Imitate this pattern**（Finger pointing "straight line"），**please draw it on this blank paper** 。

Score P（zero to one）_____★

Q. Picture design two（If the fraction Q = one, go to V, R-U give a full point）

Finger in card six“Squares containing rhombus”。**Imitate this pattern**，**please draw it on this blank paper** Picture "Squares containing diamonds"（one point）

Score Q（zero to one）_______★

1. Picture design three Finger in card seven。**Imitate this pattern**（Finger in "square and diamond"），**please draw it on this blank paper** Score R（zero to one）___★
2. Picture design four Finger in card eight。**Imitate this pattern**（Finger in "rhombus"），**please draw it on this blank paper**。 Score S（zero to one）___★

T. Picture design five Finger in card nine。**Imitate this pattern**（Finger finger "square"）。**please draw it on this blank paper**。 Score T（zero to one）___★

U. Picture design six **Please write your name**（Finger in blank paper）。 Score U（zero to one)_____★

1. Same and different: display the card ten to card seventeen in turn.**Look at these three patterns, tell me which two are the same or which two is similar?**

**Which one is different or which one is different from the other two?** Same (one point) Different (one point)

V1.card ten Same_______Different ______

V2.card eleven Same________Different_________

V3.card twelve Same_______Different ______

V4.card thirteen Same________Different_________

V5.card fourteen Same_______Different ______

V6.card fifteen Same________Different_________

V7.card sixteen Same____Different (Circular)__

V8.card seventeen Same____ Different(rectangle)_____

Score V（zero to sixteen）___________◆

*W．Approximate point (if the score W> 5, go to AA, X-Z, give full points)

**▁▁▁▁and▁▁▁▁In what ways are they similar or what are they all?** Record the answer。

W1.Apples and Bananas ________（zero to two point）

W2.Shirts and Coats_________（zero to two point）

W3.Trains and Ships _________（zero to two point）

W4.Table and chair__________（zero to two point）

Score W（zero to eight）__________◆

X。Generalization and Thinking  **Name three things you can eat/wear/transport.____，___and___In what way are they similar?...What do they have in common? Record your answers.Each correct answer =one point**

X one.Something you can eat__________________________

X two.Something to wear_______________________

X three.transport_________________________

Score X（zero to three）_______◆

Y．difference **Now I'm going to talk about three things. Please tell me which one is different from the others.Each correct answer =one point**

Y one.dog、cat、door Y two.shear、boy、girl Y three.goldfish、steamship、 train

Score Y（zero to three）__________◆

Z．Approximate point-choice question：ask：**___and___，they are______，or______，or_______？**

Z one.Apple-Banana They're all fruits（two points） they're all vegetables（one point） they're all animals（zero point）

Z two.Shirt-pants They're all clothes （two points） They're all fruit（zero point） They're all wool（one point）

Z three.ship-train They are all movable(one point)； they are all clothes（zero point）；they are all vehicles（two point）

Z four.table-chair they are all vehicles（zero point）；Both furniture（two points） They are all wood（one point）

Score Z（zero to eight）__________◆

AA．Read the sentences  **Display card eighteen。Please read the sentence out loud.Please remember it, because I will ask you to say it later.(No grading)**

AB. Spontaneous language **Please feel free to say one complete sentence.Please remember it, because I will ask you to say it later.Record sentences.**_________________（one point）

Score AB（zero to one）_______◆

AC.orienteering Each item gets one point for being correct

***Same project as MMSE**：AC one. What year is this in the Gregorian calendar? AC two. What month is it now? AC three.what is the date

AC four. what day AC five. What city is this? AC six.Where are you now?

**Add**：AC seven. Who is the current premier of China? AC eight.How old are you ?

AC nine. Where were you born?

Score AC（zero to nine）________**■**

AD.Distraction to count one Display card nineteen。（Turn the CARDS）**Count the number of "seven" and show me.**

（Subtract one point from a wrong number）

Score AD（zero to six）___________▲

AE.Distraction to count two Display card twenty.**Count the number of "seven" and show me.**

（Subtract one point from a wrong number）

Score AE（zero to five）___________▲

AF.Sentence to recall **Do you remember when I asked you to read a sentence out loud?Please tell me about it.Record sentences.**

complete sentence （four point） gray（one point）

kid （one point） dog（one point）

Score AF（zero to four）________**■**

AG.Spontaneous sentence recall **Do you remember when I asked you to say a complete sentence? Please tell me about it.Record sentences.**

complete sentence （three point）

Any word that is identical to the original sentence （one point）

Another word that is identical to the original sentence （one point）

Score AG（zero to three）________**■**

AH.Words appear how card twenty-one in the quiz kit booklet。**Please read the following words four times and try to remember each word.**

AH one. the first time Read correctly（one point） AH two. second time Read correctly（one point）

AH three. the third time Read correctly（one point） AH four. the fourth time Read correctly（one point）

Score AH（zero to four）___________▲

AI.Word recognition Show cards twenty-two to twenty-six in the test kit booklet one by one。**Now I'm going to show you some words, two at a time, and please tell me which one you just read.**（Score one point for each pair）

AI one.Twilight - Mind AI two.Size - Plants AI three.land-swich AI four.Machine - Night

AI five.Spark - Milk

Score AI（zero to five）________**■**

AJ.Visual matching Show cards twenty-seven and twenty-eight in the test kit booklet。**The pattern on my card (pointing to card twenty-eight) is exactly the same as that on this card (pointing to card twenty-seven).When I point out the design on my card, I want you to show me the same design on your card.Which pattern is the same as this one (from the subject's point of view, pointing at the top left corner of card** **twenty-seven)?**In any order, match the other three patterns. Repeat three times.

AJ1. the first time Match the correct （one point）

AJ2. second time Match the correct（one point）

AJ3. the third time Match the correct（one point）

AJ4. the fourth time Match the correct（one point） Score AJ（zero to four）___________▲

AK.Visual recognition **Now I will show you some patterns, two at a time, and please tell me which pattern you saw just now.Show cards twenty-nine to thirty-two in the test kit booklet one by one.**

AK one.left （one point） AK three.right （one point）

AK two.right （one point） AK four.left （one point）

Score AK（zero to four）__________**■**

**One．Rey-O picture,imitation**"I'll show you a picture now.Please draw that picture on white paper ".(Record time, do not forecast to recall.Start three strokes with the rest of the lines using a different color pen or qualitative type.

**Two．The logical memory-I（Read the story and recall it immediately）**"Now please read a story and then please retell it."Don't tell me to remember again.Please record the wrong answers.）

**Three.digit span:** instruction：I say some figures, you listen carefully, when I have finished, you repeat the same (read at a constant rate of one number per second) record the points： Recite in order + Reverse order to recite =

Recite in order Reverse order to recite

| three. Five to eight to two | two. Two to four |
| --- | --- |
| . Six to nine to four | Five to eight |
| Four. six to three to nine | Three. six to two nine |
| Seven to two to eight to six | Four to one to five |
| Five. Four to seven to three to one | Four. three to two to seven to nine |
| seven to five to eught to three to six | Four to nine to six to two |
| Six. Six to one to nine to four to seven to three | Five. one to five to two to eught to six |
| three to nine to two to four to eight to seven | Six to one to eughtto four to three |
| Seven. five to nine to seven to one to four to two to eight | Six. five to three to nine to four to one to eight |
| four to one to seven to nine to three to eight to six | seven to two to four to eight to five to six |
| Eight. five to eight to one to nine to two to four to six to seven | seven eight to one to two to nine to three to six to five |
| three to eight to two to nine to five to one to seven to four | four to seven to three to nine to one to two to three |
| nine two to seven to five to eight to six to two to five to eight to four | Eight. nine to four to three to seven to six to two to to five to eight |
| seven to one to three to nine to four to two to five to six to eight | seven to two to eight to one to nine to six to five to three |
| Ten. five to two to seven to four to nine to one to three to seven to four to six | Nine. six to three to one to nine to four to three to six to five to eight |
| four to seven to two to five to nine to one to six to two to five to three | nine to four to one to five to three to eight to five to seven to two |
| eleven four to one to six to three to eight to two to four to six to three to five to nine | ten six to four to five to two to six to seven to nine to three to eight to six |
| three to six to one to four to nine to seven to five to one to four to two to seven | five to one to six to two to seven to four to three to eight to five to nine |
| twelve seven to four to nine to six to one to three to five to nine to six to eight to two to five |  |
| six to nine to four to seven to one to nine to seven to four to two to five to nine to two |  |

**Four.Similarity test: record false answers.**

| content | answer | grade | content | answer | grade |
| --- | --- | --- | --- | --- | --- |
| one.Axe to Saw |  |  | two．The dog to lion |  |  |
| three．Orange to peach |  |  | four．Table to chair |  |  |
| five．Hat and socks |  |  | six．Egg-seeds |  |  |
| seven．North to West |  |  | eight．Eye to ear |  |  |
| nine．Air and water |  |  | ten．Poems to Statues |  |  |
| eleven．Praise and Punishment |  |  | twelve．Wood to alcohol |  |  |
| thirteen．The fly to tree |  |  |  |  |  |

**five. Stroop test**

Card A Instructions:**"From left to right, please read the following Chinese characters as quickly and correctly as possible."**

| yellow | red | blue | yellow | green | red | blue | red | blue | yellow |  |
| --- | --- | --- | --- | --- | --- | --- | --- | --- | --- | --- |
| blue | yellow | yellow | blue | red | blue | yellow | green | green | red |  |
| red | green | green | red | green | green | green | yellow | red | green |  |
| green | blue | blue | yellow | yellow | yellow | red | red | yellow | green |  |
| yellow | red | green | yellow | blue | green | red | green | green | blue |  |

卡片B Instructions:**"From left to right, please read the names of the following colors as quickly and correctly as possible."**

| blue | green | red | blue | yellow | green | yellow | blue | yellow | red |  |
| --- | --- | --- | --- | --- | --- | --- | --- | --- | --- | --- |
| green | blue | green | red | green | yellow | blue | red | blue | yellow |  |
| blue | red | blue | green | red | yellow | red | blue | green | yellow |  |
| red | yellow | red | blue | green | blue | green | yellow | blue | yellow |  |
| red | blue | yellow | red | green | blue | yellow | red | blue | yellow |  |

Card C Instructions: "From left to right, please read the names of the following colors as quickly and correctly as possible, not the words.For example, the first one is pronounced "green" instead of orchid .Got it?now

| green | yellow | blue | green | red | yellow | blue | red | blue | green |  |
| --- | --- | --- | --- | --- | --- | --- | --- | --- | --- | --- |
| blue | red | green | blue | yellow | red | green | yellow | red | blue |  |
| red | blue | yellow | red | blue | green | yellow | red | yellow | green |  |
| blue | red | green | yellow | red | yellow | blue | green | red | green |  |
| yellow | red | blue | green | blue | green | red | blue | yellow | red |  |

| Index | Card one | Card two | Card three |
| --- | --- | --- | --- |
| time（second） |  |  |  |
| Number of correct readings（M=fifty） |  |  |  |

**six.Rey-O picture, Recall "Please draw the picture you have just drawn on white paper again."(Unlimited time.Use blue for the first four strokes and black for the rest of the lines)**

**seven．Logical memory-II (story again, "now, please recall the story you just read again. Not limited. Notice No need to recall again.)**

**eight. Audition word learning test（N1、N2、N3）**

| No | project | N1 | N2 | N3 | After  five  minutes | N4 | After  twenty  minutes | N5 | N six |  | Once again | |
| --- | --- | --- | --- | --- | --- | --- | --- | --- | --- | --- | --- | --- |
| one | coat |  |  |  |  |  |  |  | Flowers in class | wintersweet | R soldier | R  pants |
| two | The driver |  |  |  |  |  |  |  |  | Chinese flowering crabapple | button | R  gloves |
| three | Chinese flowering crabapple |  |  |  |  |  |  |  |  | yulan | R  Chinese flowering crabapple | soldier |
| four | woodworking |  |  |  |  |  |  |  |  | lily | business suit | R  chinese flowering crabapple |
| five | trousers |  |  |  |  |  |  |  | Professional class | lawyer | earrings | cuckoo |
| six | lily |  |  |  |  |  |  |  |  | driver | R  yulan | R carpenter |
| seven | scarf |  |  |  |  |  |  |  |  | soldiers | director | peony |
| eight | wintersweet |  |  |  |  |  |  |  |  | woodworking | lotus | R  coat |
| nine | The soldiers |  |  |  |  |  |  |  | apparel | pants | R  kerchief | shirt |
| ten | yulan |  |  |  |  |  |  |  |  | gloves | R  driver | R  lawyer |
| eleven | The lawyer |  |  |  |  |  |  |  |  | kerchief | leather shoes | schoolmaster |
| twelve | gloves |  |  |  |  |  |  |  |  | coat | corn | R  wintersweet |
| correct |  |  |  |  |  |  |  |  |  |  |  |  |
| Insert error |  |  |  |  |  |  |  |  |  |  |  |  |
|  |  |  |  |  |  |  |  |  |  |  |  |  |
|  |  |  |  |  |  |  |  |  |  |  |  |  |

When the subjects answered "coat, woodwork, shirt, woodwork", the order was recorded as:one coat ,two woodwork , three shirt , four woodwork .Record as much as possible.

1. **The attachment test**

| project | Trails1  exercises | Trails1  quizzes | Trails2  exercises | Trails2  quizzes |
| --- | --- | --- | --- | --- |
| timing |  |  |  |  |
| Number of notifications of an incorrect connection |  |  |  |  |
| The number of times I raised my pen to remind you |  |  |  |  |

**Ten.Auditory word learning test（N4）**

**eleven.Word fluency**

List the number of animals

| **One to fifteen seconds** | **Sixteen to thirty seconds** |
| --- | --- |
| **thirty-one to forty-five seconds** | **forty-six to sixty seconds** |

**The number of fruit**

| **One to fifteen seconds** | **Sixteen to thirty seconds** |
| --- | --- |
| **thirty-one to forty-five seconds** | **forty-six to sixty seconds** |

**Number of vegetables**

| **One to fifteen seconds** | **Sixteen to thirty seconds** |
| --- | --- |
| **thirty-one to forty-five seconds** | **forty-six to sixty seconds** |

**twelve. Clock Search Test**

**Step one: The person performing the test points to fifteen items in Figure one and asks for names. If the person tested can't name a certain item, he can tell him directly.Step two: The person performing the test puts Picture two in front of the subject and says, "Please circle all the small clocks you find."Step three: After you're done, "you double-check to see if you missed anything."he can use a red pen to tell the difference.**

| bird | apple | cloud | guitar | house |
| --- | --- | --- | --- | --- |
| automobile | key | Small bell | gun | tree |
| horse | fish | table lamp | teapot | saw |

|  | spontaneous | After the advice |
| --- | --- | --- |
| Correct number | ( )( )( )( )( )( )( ) | ( )( )( )( )( )( )( ) |
| Completion time |  |  |
| Wrong number |  |  |

**Thirteen.Clinical Dementia Scale (CDR)**

| Function | Normal  CDR=0 | suspicious  CDR=0.5 | [mild](../../../../E:/Program%20Files%20(x86)/Youdao/Dict/8.9.6.0/resultui/html/index.html#/javascript:;)  CDR=1 | [moderate](../../../../E:/Program%20Files%20(x86)/Youdao/Dict/8.9.6.0/resultui/html/index.html#/javascript:;)  CDR=2 | Severe  CDR=3 |
| --- | --- | --- | --- | --- | --- |
| [memory](../../../../E:/Program%20Files%20(x86)/Youdao/Dict/8.9.6.0/resultui/html/index.html#/javascript:;) | No memory loss or slight intermittent amnesia | Mild persistent forgetfulness;Partial recall of events;"Benign amnesia" | Moderate memory impairment;Recent amnesia is more obvious;Daily life is affected | Severe memory impairment;Save only very familiar materials;New material is quickly forgotten | Severe memory impairment;Save only fragment material |
| [directive](../../../../E:/Program%20Files%20(x86)/Youdao/Dict/8.9.6.0/resultui/html/index.html#/javascript:;) [force](../../../../E:/Program%20Files%20(x86)/Youdao/Dict/8.9.6.0/resultui/html/index.html#/javascript:;) | Directional complete | Except for time orientation that is sometimes slightly difficult, orientation is complete | Time oriented moderate damage;There may be geo-orientation errors | Severe temporal disorientation;There is often location disorientation | save only character orientation |
| Determine and solve problems | Able to solve daily problems and money transactions well;Determine the correct | Problem solving ability and judging similarities and differences between things were mildly impaired | Moderate impairment in problem-solving ability and judgment of similarities and differences;Social judgments usually remain | The ability to solve problems and judge similarities and differences is seriously impaired;Social judgment is often impaired | Inability to make judgments and solve problems |
| Work and social skills | Usual work, shopping and social functions are normal | These activities are mildly impair | engaged but cannot perform these activities on their own;Careless examination showed normal performance | Loss of social skills;You can still do some outdoor activities | Inability to play outdoor activities |
| Family life and hobbies | Maintain a good family life, hobbies and interests | Slight impairment of family life, hobbies and interests | Mild but certain damage to family life;Give up more difficult chores and more complex hobbies and interests | Can only complete simple housework;Very limited interest | no apparent ability to live at home |
| Ability to live independently | Independent living | Totally able to take care of himself | Need to be prodded | Need help with dressing and personal hygiene | Needs a lot of help taking care of;Incontinence in stool and urine |

**fourteen．Boston naming Quiz: Instructor: "I'm going to show you some pictures. Please tell me what they are."Answer and identify without prompting.Record the actual answers.**

**fifteen.Draw the clock test**

**Draw the clock "1:50" on blank paper.Use blue for the first 4 strokes, black for the rest of the lines, and red for the supplement and modification of the standard clock.**

| **Project** | **[full](../../../../E:/Program%20Files%20(x86)/Youdao/Dict/8.9.6.0/resultui/html/index.html#/javascript:;) [mark](../../../../E:/Program%20Files%20(x86)/Youdao/Dict/8.9.6.0/resultui/html/index.html#/javascript:;)** |  |  |  |
| --- | --- | --- | --- | --- |
| one.Anchor 12,3,6,9 | four |  |  |  |
| two.Write down all the numbers | four |  |  |  |
| three.All numbers are in the circle of the clock face | two |  |  |  |
| four.Arrange clockwise | one |  |  |  |
| five.one-twelve Number order | one |  |  |  |
| six."twelve,three,six,nine" are symmetrical | two |  |  |  |
| seven.The position of the other eight numbers | three |  |  |  |
| eight.Central location | one |  |  |  |
| nine.Dial | one |  |  |  |
| 10.The hour hand and minute hand | two |  |  |  |
| eleven.Clockwise | two |  |  |  |
| twelve.The minute hand points | two |  |  |  |
| thirteen.Pointer length | one |  |  |  |
| fourteen.The minute hand is longer than the hour hand | one |  |  |  |
| fifteen.A minute hand is thinner than an hour hand | one |  |  |  |
| sixteen. Both the hour hand and the minute hand have arrows | two |  |  |  |

**sixteen．Auditory word learning test（N5、N6、recognition）**

**seventeen.Bristol Daily Living Ability Scale**

(For those in the know) Think back to the last two weeks and circle the number that represents your relative or friend's ability to act accordingly.Only one number can be circled for each action.(If in doubt, select an ability level that represents the average of the last 2 weeks.)A score of 0 is completely normal, and a score of 3 is not at all.

| Item | zero | one | two | three | Item | zero | one | two | three |
| --- | --- | --- | --- | --- | --- | --- | --- | --- | --- |
| One.cook meals |  |  |  |  | Eleven.walk |  |  |  |  |
| Two. meals |  |  |  |  | Twelve.time orientation |  |  |  |  |
| Three.Prepare drinks |  |  |  |  | Thirteen.space oriented |  |  |  |  |
| Four..Have a drink |  |  |  |  | Fourteen.Conversation |  |  |  |  |
| Five. Dress |  |  |  |  | Fifteen.Make a phone call |  |  |  |  |
| Six.Hygiene |  |  |  |  | Sixteen.Do housework |  |  |  |  |
| Seven. Brush your teeth |  |  |  |  | Seventeen.Go shopping |  |  |  |  |
| Eight.Take a bath |  |  |  |  | Eighteen.Manage your money |  |  |  |  |
| Nine. Going to the toilet |  |  |  |  | Nineteen. Hobbies |  |  |  |  |
| Ten. move |  |  |  |  | Twenty.vehicles |  |  |  |  |

**Self-rating Depression Scale was used to investigate the prevalence of depression（CES－D）**

Explanation: Here are some situations or thoughts that you may have had or felt.Please tick "√" in the appropriate box according to your actual situation or feeling during the past week:

zero points = none or almost none (no more than one day in the past week)

one points = rarely (one to two days in the past week)

two points = often (three to four days in the past week)

three points = almost all the time (six to seven days in the past week)

|  | none or almost none | rarely | Often | Almost all the time |
| --- | --- | --- | --- | --- |
| One.I worry about some trifles |  |  |  |  |
| Two.I don't really want to eat. I have a bad appetite |  |  |  |  |
| Three.Even if my family and friends help me, I still can't get rid of the depression in my heart |  |  |  |  |
| Four.I don't think I'm as good as average people |  |  |  |  |
| Five.I can't concentrate when I'm doing something |  |  |  |  |
| Six.I'm feeling low |  |  |  |  |
| Seven. I find it hard to do anything |  |  |  |  |
| Eight. I feel hopeless about my future |  |  |  |  |
| Nine. I feel that my life is a failure |  |  |  |  |
| Ten.I feel afraid |  |  |  |  |
| Eleven.I'm not sleeping well |  |  |  |  |
| Twelve.I can't feel happy |  |  |  |  |
| Thirteen.I talk less than usual |  |  |  |  |
| Fourteen.I feel lonely |  |  |  |  |
| Fifteen.I don't think people are very friendly to me |  |  |  |  |
| Sixteen.I don't think life is interesting |  |  |  |  |
| Seventeen.I've cried |  |  |  |  |
| Eighteen.I feel sad |  |  |  |  |
| ninteen.I don't think people like me |  |  |  |  |
| twenty.I don't feel able to go on with my daily work |  |  |  |  |

**Results: The main statistical index was the total score.≤fifteen was considered as having no depressive symptoms;sixteen to ninteen were likely to have depressive symptoms;A score greater than or equal to twenty must have depressive symptoms.**

**Eighteen.The Geriatric Depression Scale (GDS) selects the answer that best fits your feelings during the week, selecting "Yes" or "No" after each question.**

**(one) Fill in by the subject;**

**(two)one, five, seven, nine, fifteen, ninteen, twenty-one, twenty-seven, twenty-nine, thirty are inverted questions, the answer "no" will get one point;Anyone who answers "yes" to each of the other questions gets one point.**

| one | Are you generally satisfied with your life? | Yes No |
| --- | --- | --- |
| two | Have you given up many of your hobbies and interests? | Yes No |
| three | Do you feel your life is empty? | Yes No |
| four | Are you bored? | Yes No |
| five | Do you see any hope for the future? | Yes No |
| six | Are you bothered by thoughts that you can't get out of your head? | Yes No |
| seven | Are you energized most of the time? | Yes No |
| eight | Are you afraid that something bad will happen to you? | Yes No |
| nine | Are you happy most of the time? | Yes No |
| ten | Do you often feel alone? | Yes No |
| eleven | Do you often fidget and get upset? | 是 否 |
| twelve | Would you rather stay at home than do something new outside? | Yes No |
| thirteen | Do you often worry about your future life? | Yes No |
| fourteen | Do you think your memory is worse than before? | Yes No |
| fifteen | Are you happy to be alive right now? | Yes No |
| sixteen | Do you often feel heavy and depressed? | Yes No |
| seventeen | Do you feel there is no point in living as you are? | Yes No |
| eighteen | Do you always worry about things that are past? | Yes No |
| ninteen | Do you find life exciting? | Yes No |
| twenty | Do you have a hard time starting a new project? | Yes No |
| Twenty-one | Do you feel alive? | Yes No |
| Twenty-two | Do you feel that your situation is hopeless? | Yes No |
| Twenty-three | Do you feel that most people are much better than you? | Yes No |
| Twenty-four | Do you often sad about the little things? | Yes No |
| Twenty-five | Do you often feel like crying? | Yes No |
| Twenty-six | Do you have trouble concentrating? | Yes No |
| Twenty-seven | Do you enjoy your morning? | Yes No |
| Twenty-eight | Do you want to avoid parties? | Yes No |
| Twenty-nine | Do you find it easy to make your decisions? | Yes No |
| thirty | Is your mind as clear as usual? | Yes No |

**Rey-O picture,imitation**"I'll show you a picture now.Please draw that picture on white paper."

(one）Record time without limiting time or pressing

(two).qualitative analysis：type Ⅰ：Draw the center rectangle frame first and fill in the details； type Ⅱ：Draw some details and rectangles first and then fill in the rest； type Ⅲ：Without clearly distinguishing the rectangular frame, draw the outline first and then fill in the details；type Ⅳ：The details are pieced together into a complete picture；type Ⅴ：You can only draw some details, you can't tell the whole picture； type Ⅵ：Simplify to general figures such as houses, boats, etc； type Ⅶ：It's not relevant to the original picture。

Trail Making Test(Trail Making Test，TMT)

operating steps：exercises：Here are some numbers, and when I say "go", please put them together in order.From one(pointing to 1), to two(pointing to two), to three(pointing to three)...And so on until you're done.notice: **Keep the nib of the pen on the paper.The lines you draw must go through the figure.Now please put your pen here (pointing to the starting point)**。When I say "start", you connect the numbers in order as fast as you can until you get to the end.Are you ready?Start.(Timing).

quizzes：Here are some more numbers, so just put them together in the same order as before.The nib must not leave the paper and the lines must be drawn through the figures.Start here (pointing to the starting point) and end here (pointing to the ending point).Are you ready?Start.(Timing).
